# Polymerized-Type I Collagen Downregulates Inflammation and Improves Clinical Outcomes in Patients with Symptomatic Knee Osteoarthritis Following Arthroscopic Lavage: A Randomized, Double-Blind, and Placebo-Controlled Clinical Trial

**DOI:** 10.1100/2012/342854

**Published:** 2012-04-01

**Authors:** Janette Furuzawa-Carballeda, Guadalupe Lima, Luis Llorente, Carlos Nuñez-Álvarez, Blanca H. Ruiz-Ordaz, Santiago Echevarría-Zuno, Virgilio Hernández-Cuevas

**Affiliations:** ^1^Department of Immunology and Rheumatology, Instituto Nacional de Ciencias Médicas y Nutrición Salvador Zubirán, Vasco de Quiroga No. 15, Col Sección XVI, 14000 Mexico City, DF, Mexico; ^2^Department of Molecular Biology and Biotechnology, Instituto de Investigaciones Biomédicas, Universidad Nacional Autónoma de México, Apartado Postal 04510, México City, DF, Mexico; ^3^Unidad Médica de Alta Especialidad, Hospital de Traumatología y Ortopedia, IMSS, Boulevard Manuel Ávila Camacho s/n, Ex-ejido de Oro, 53120 Naucalpan, MEX, Mexico

## Abstract

*Objectives*. Polymerized-type I collagen (polymerized collagen) is a downmodulator of inflammation and cartilage regenerator biodrug. *Aim*. To evaluate the effect of intraarticular injections of polymerized collagen after arthroscopic lavage on inflammation and clinical improvement in patients with knee osteoarthritis (OA). *Methods*. Patients (*n* = 19) were treated with 6 intraarticular injections of 2 mL of polymerized collagen (*n* = 10) or 2 mL of placebo (*n* = 9) during 3 months. Followup was 3 months. The primary endpoints included Lequesne index, pain on a visual analogue scale (VAS), WOMAC, analgesic usage, the number of Tregs and proinflammatory/anti-inflammatory cytokine-expressing peripheral cells. Secondary outcomes were Likert score and drug evaluation. Clinical and immunological improvement was determined if the decrease in pain exceeds 20 mm on a VAS, 20% of clinical outcomes, and inflammatory parameters from baseline. Urinary levels of C-terminal crosslinking telopeptide of collagen type II (CTXII) and erythrocyte sedimentation rate (ESR) were determined. *Results*. Polymerized collagen was safe and well tolerated. Patients had a statistically significant improvement (*P* < 0.05) from baseline versus polymerized collagen and versus placebo at 6 months on Lequesne index, VAS, ESR, Tregs IL-1*β*, and IL-10 peripheral-expressing cells. Urinary levels of CTXII were decreased 44% in polymerized collagen versus placebo. No differences were found on incidence of adverse events between groups. *Conclusion*. Polymerized collagen is safe and effective on downregulation of inflammation in patients with knee OA.

## 1. Introduction

Osteoarthritis (OA), the most common form of arthritis, is generally considered as a degenerative disorder. The incidence of knee OA is high in the years following anterior cruciate ligament or meniscal injury, and evidence suggests that current arthroscopic procedures, including reconstruction of the anterior cruciate ligament and meniscectomy, are not sufficient to restore normal joint mechanics or neutralize the long-term risk of OA [1]. On the other hand, OA can be viewed as an inflammatory disease characterized by progressive deterioration of articular cartilage and synovial joints [2–5]. Interleukin (IL)-1*β*, tumor necrosis factor (TNF)-*α*, and IL-6 seem to be the main proinflammatory cytokines involved in the pathophysiology of OA, even though others, including IL-15, IL-18, IL-21, leukemia inhibitory factor (LIF), and chemokines have also been implicated [6]. Recent progress has considerably improved knowledge of both the factors involved in the development of OA and the mechanisms responsible for its progression. Therefore, a new therapeutic strategy is to develop drugs capable of modifying the structural progression of OA (disease-modifying OA drugs or DMOADs) in order to ameliorate the effect of increasing OA prevalence. DMOADs can cause retardation of disease progression, a complete halt in disease progression, regeneration of cartilage, and even the prevention of disease development. DMOADs in phase II/III clinical development include oral salmon calcitonin, SD-6010, vitamin D3 (cholecalciferol), collagen hydrolysate, recombinant human fibroblast growth factor (FGF)-18, bone morphogenetic protein (BMP)-7, avocado-soybean unsaponifiable (ASU), and polymerized-type I collagen (ASPID PHARMA SA de CV, Mexico City, Mexico) [7–9].

Polymerized-type I collagen is a *γ*-irradiated mixture of pepsinized porcine type I collagen and polyvinylpyrrolidone. The addition of 1% polymerized-type I collagen to cartilage and synovial tissue cocultures has shown to induce cartilage regeneration owing to an increase of 3- to 6-fold chondrocytes proliferation (Ki-67) and cartilage extracellular matrix proteins (proteoglycans, cartilage oligomeric matrix protein or COMP, and type II collagen). Moreover, polymerized-type I collagen induced downmodulation of inflammation inhibiting proinflammatory cytokine expression (IL-1*β* and TNF-*α*) and inducing upregulation of IL-10, an anti-inflammatory cytokine. No differences were found on IL-8 or TIMP-1 levels in supernatants from Polymerized-collagen-treated cocultures when compared with untreated cultures. Meanwhile IL-12 and IFN-*γ* were undetectable [8]. Intraarticular (IA) administration of 2 mL of polymerized type I to knee OA patients showed a statistically significant improvement in Lequesne Index, WOMAC, pain intensity on a visual analogue scale (VAS), patient global score, and analgesic usage. This improvement was persistent during the followup [9].

We consider that the administration of pharmacologic agents at critical times, such as following injury and perioperatively might prevent disease development. For this reason, the aim of the study was to evaluate the effect of IA injections of polymerized collagen, on inflammation and clinical improvement in patients with knee OA after arthroscopic lavage.

## 2. Materials and Methods

### 2.1. Trial Design

This was a prospective, randomized, double-blind, placebo controlled clinical trial.

### 2.2. Study Population

#### 2.2.1. Inclusion Criteria

The protocol was approved by the IMSS Committee of Medical Ethics (Ref. no. 2800-758-053) and was performed in accordance with the revised Declaration of Helsinki, 1983. Only patients who gave written informed consent to participate were recruited. Patients who fulfilled the 1986 American College of Rheumatology for the classification of knee OA were included [10].

#### 2.2.2. Exclusion Criteria

Patients who received oral, IA, or parenteral corticosteroid use within 3 months, IA injection of any hyaluronic substance into the knee within 90 days, or operative arthroscopy within 5 months or treatment with anticoagulants were excluded of the study. Patients with concurrent medical or arthritic conditions that could interfere with evaluation of the index knee joint, including fibromyalgia, Reiter's syndrome, rheumatoid arthritis, psoriatic arthritis, ankylosing spondylitis, lymphoma, arthritis associated with inflammatory bowel disease, sarcoidosis, amyloidosis, clinical signs and symptoms of active knee infection, crystal disease, cancer, more significant pain from the back or the hip than the knee, patients with HIV or HCV, and patients with drug or alcohol dependence history or sensitivity to polymerized-type I collagen were also excluded.

### 2.3. Study Protocol

We calculated the sample size of 9 per group. Patients were allocated using a random number generation and block randomization to two parallel groups [11].

Nineteen patients with a body mass index (BMI) ≤ 40 kg m^−2^, on stable therapy with NSAIDs and negative to a standard forearm skin test to polymerized collagen administration (0.2 mL of polymerized collagen at 72 h of the initial skin challenge) were enrolled in a 6-month study (1-week run-in phase, 12-week treatment phase, and 3-month follow-up phase). At baseline visit the eligibility of patients for the study was confirmed by review of history, clinical examination of the knee to be treated, and laboratory tests. Blood was taken for hematology and clinical chemistry assessments at baseline, 3, and 6 months. Patients were provided with instructions on a set of standard physiotherapy exercises to be performed throughout the study. Patients were instructed in the daily use of a diary card on which it was recorded compliance with standard physiotherapy and the use of any additional analgesia or NSAIDs. Patients were also asked to record adverse events (any unwanted event occurring during the course of the trial whether if it was considered to be related to administration of the study biodrug). At each subsequent visit, efficacy evaluations were conducted, and adverse events and concomitant medications were recorded prior to administration of study medication.

#### 2.3.1. Arthroscopic Lavage

Arthroscopic lavage, with or without debridement, was performed in both groups. Briefly, skin around the knee was cleaned with a povidone-iodine solution; this was followed by an injection of local anesthetic, into the outer mediopatellar zone. The anesthetic was allowed to act, and an access way was then opened with number 6 abocat. The administration of the saline lavage was preceded by drainage of any effusion in the joint in order to evacuate it as thoroughly as possible. Then, a volume around 100 cm^3^ of cold saline was instilled through the outer access way. Arthroscopic partial meniscectomy or loose body removal was performed. Once the knee was distended, local anesthetic was injected into the inner mediopatellar zone, and a new abocat guide was used to establish the inner drainage way, similarly to the outer one. The lavage proper involved the instillation of 3 L of cold (8°C) saline at a constant flow rate by using a dropper line connected to the entry way; the inner zone was also connected to another, free-fall dropper line that ended in a biological sample container. The perfusion time ranged from 90 to 120 min, depending on the individual characteristics of the patients. Once perfusion was completed, any fluid remaining in the joint was evacuated by manually squeezing the distended joint cavity.

#### 2.3.2. Intervention

After lavage, 2 mL of polymerized collagen (13.8 mg of collagen) or 2 mL of placebo (PVP citric/citrate buffer) were administered into the joint under direct ultrasound to ensure intraarticular injection. A compression bandage was applied to the leg. It was removed by the patient 24 h later. Six weeks after surgery patients received 5 IA injections of 2 mL of polymerized collagen or 2 mL of placebo (one per week) by either lateral or medial approach after the instillation of 1 mL of 1% xylocaine solution at weeks 8, 9, 10, 11, and 12 in the same knee that was selected by the physician. The two experimental preparations were visually identical, and its viscosity was very similar. Handling and preparation of polymerized-type I collagen and placebo were carried out by IMSS staff in such a way as to maintain study blinding, except that the study pharmacist was unblinded to each participant's study treatment. The institutional pharmacists had access to a code list identifying whether the participant received polymerized-type I collagen or placebo. The pharmacist did not disclose this information to any study personnel. The participant study site personnel (other than the research pharmacist) and patient were all blinded to the treatment assignments. The rationale to inject IA polymerized collagen was based on the previous experience related to the biodrug effects (anti-inflammatory and tissue regenerator effects) observed during the course of the treatment of knee OA [8, 9, 12–14].

### 2.4. Clinical Interview and Outcomes

Clinical evaluation was performed at baseline and every month during the study. The primary endpoint included pain intensity on 10 mm VAS (patient and physician), WOMAC instrument [15], Lequesne index [16], and analgesic consumption. Secondary outcome measures included patient's and investigator's global assessment of disease activity on a 5-point rating scale, global assessment of change in disease activity at the end of the treatment (Likert score: 0 = very poor; 1 = poor; 2 = fair; 3 = well; 4 = very well) and patient's and physician's response to therapy (evaluation of medication: 0 = none: no good at all, ineffective drug; 1 = poor: some effect, but unsatisfactory; 2 = fair: reasonable effect, but could be better; 3 = good: satisfactory effect with occasional episodes of pain or stiffness; 4 = excellent: ideal response, virtually pain-free) [17]. Clinically significant improvement was determined if the decrease in pain exceeds 20 mm on the 0–100 mm VAS [18] and patients achieved at least 20% of improvement from baseline in WOMAC [16, 18, 19] and Lequesne Index [17].

### 2.5. Safety Assessment

The safety of treatment was determined from the occurrence of systemic and local adverse events. Adverse events and serious adverse events were assessed by the investigator at each visit and followed until resolution. Safety monitoring included records of vital signs and clinical laboratory tests (blood chemistry, urinalysis and liver function tests).

### 2.6. Laboratory Tests

Urinary levels of C-terminal cross-linking telopeptide of collagen type II (CTXII) were assessed by a one-step sandwich enzyme immunoassay (ELISA) (Urine CartiLaps EIA, Immunodiagnostic Systems Inc, Arizona, USA). The CTX-II values were corrected with creatinine concentration (*μ*g mmol^−1^) and perform the correction using the equation: corrected CTX-II value (ng mmol^−1^) = 1000 × Urine CartiLaps (*μ*g L^−1^)/creatinine (mmol L^−1^) [19]. Erythrocyte sedimentation rate (ESR) was determined by Westergren method. Anticyclic citrullinated peptide (anti-CCP3) IgG antibodies were determined with a commercial ELISA kit (INOVA, San Diego, CA). Assay was performed according to the manufacturer's instructions.

### 2.7. Peripheral Blood Mononuclear Cells (PBMCs) Isolation

A 10 mL sample of venous blood was obtained from each subject under polymerized collagen or placebo treatment at baseline, 3, and 6 months of the study. Thirteen healthy age-matched subjects without overweight (in order to avoid systemic inflammation associated with body fat) who volunteered were included as controls. PBMCs were obtained by gradient centrifugation on Lymphoprep (Axis-Shield PoC AS, Oslo, Norway).

### 2.8. Flow Cytometry

PBMCs (1×10^6^) were stained with 5 *μ*L of anti-CD4-PECy5-labelled, anti-CD14-FITC-labelled, anti-CD8-PECy5-labelled, and anti-CD28-FITC-labelled monoclonal antibodies (BD Biosciences, San Jose, CA) at room temperature in the dark for 20 min. After two washes, PBMC were permeabilized with 200 *μ*L of cytofix/cytoperm solution (BD Biosciences) at 4°C for 20 min. PBMCs were stained for intracellular cytokines and transcription factors with anti-IL-1*β*-PE-labelled, anti-TNF-*α*-PE-labeled, anti-IL-10-PE-labelled, anti-IFN-*γ*-PE-labeled (BD Biosciences), and PE-labelled-anti-Foxp3 for Tregs (eBioscience) monoclonal antibodies, for 30 min at 4°C in the dark. Finally, after washing, PBMCs subsets were analyzed by flow cytometry with a FACScan (BD Biosciences). An electronic gate was made for CD4+/CD14−, CD4−/CD14+, CD8+/CD28−, or CD8+ cells, and a total of 50000 events were recorded for each sample and analyzed with the CellQuest software (BD Biosciences). Results are expressed as the relative percentage of IL-1*β*, TNF-*α*, IL-10, IFN-*γ*, or Foxp3-expressing cells in each gate. As isotype controls, IgG_1_-FITC/IgG_1_-PE/CD45-PeCy5 mouse IgG_1_k (BD Tritest, BD Biosciences) were used to set the threshold and gates in the cytometer.

In order to avoid false positive PE results and also for setting compensation for multicolor flow cytometric analysis, we performed instrument calibration/standardization procedures each day according to established protocols of our laboratory. Briefly, we run an unstained (autofluorescence control) and permeabilized PBMCs sample. Autofluorescence control (unstained cells) was compared with single stained cell positive controls to confirm that the stained cells were on scale for each parameter. Besides, BD Calibrite 3 beads were used to adjust instrument settings, set fluorescence compensation, and check instrument sensitivity (BD CaliBRITE, BD Biosciences).

### 2.9. Statistical Analysis

We calculated the sample size with a formula for calculating sample for proportion: *N* = [√{*pq*(1 + 1/*k*)}*z*_1_ − *α*/2 + √{*p*_1_ *q*_1_ + *p*_2_ *q*_2_/*k*}*z*_1−*β*_]^2^/Δ^2^. We considered a 20% dropout rate. For the primary analysis, the means of the scores were compared between the two treatment groups on an intention to treat (ITT) basis (all patients who received a dose of study medication and had at least one efficacy observation recorded after treatment). Statistical analysis was performed using the SigmaStat11 program by One Way Analysis of Variance on Ranks and by Holm-Sidak method for all pairwise multiple comparison procedures. Data were expressed as the mean ± SD. Values smaller than or equal to 0.05 were considered as significant.

## 3. Results

Nineteen patients who met criteria for inclusion into the study were randomized to polymerized collagen (*n* = 10) or placebo (*n* = 9) treatments; all patients completed the study.

### 3.1. Patient Demographics and Baseline Disease Characteristics

Sixty seven per cent in placebo and 80% in polymerized collagen group were females. The two treatment groups were similar with respect to age, disease duration, and BMI, with no statistical differences (Table 1).

### 3.2. Primary Clinical Outcomes

**Figure 1:**
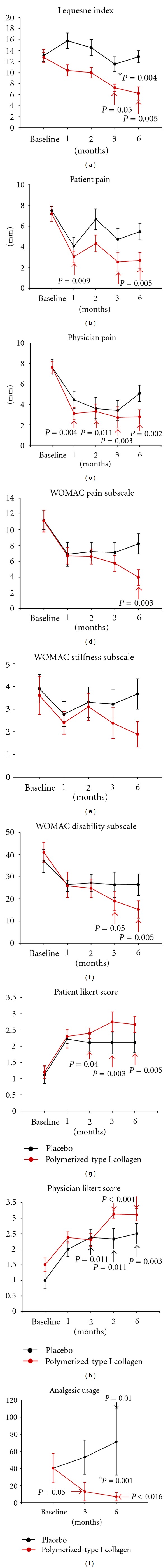
Primary and secondary measures of efficacy. Clinical evaluation was performed at baseline and every month during the study. The primary endpoints included (a) Lequesne index, (b) patient pain visual analogue scale (VAS), (c) physician pain visual analogue scale (VAS), (d) WOMAC pain subscale, (e) WOMAC stiffness subscale, (f) WOMAC disability subscale, (g) patient's and (h) physician's global assessment of disease activity on a 5-point rating scale, global assessment of change in disease activity at the end of the treatment (Likert score: 0 = very poor; 1 = poor; 2 = fair; 3 = well; 4 = very well), (i) consumption of NSAIDs tablets per month. Arrows depict the month in which the treatment reached a *P* < 0.05 compared to baseline, in black for placebo and in red for polymerized collagen group. Results represent mean ± SD. *P* values indicate statistical significant differences between treatment groups.

Scores decreased at statistically significant levels from baseline to 3 months (treatment phase) and 6 months (followup) for patients under Polymerized-type I collagen treatment compared with patients under placebo treatment (Table 2, Figure 1), in Lequesne Index (*≈*−43% and *≈*−51% at treatment phase and followup; Figure 1(a)), WOMAC pain subscale *≈*−51% at 6 months; Figure 1(d)), WOMAC stiffness subscale *≈*−49% at 6 months; Figure 1(e)), WOMAC disability subscale (*≈*−28%, and *≈*−42% at 3 and 6 months; Figure 1(f)), patient pain on a VAS (*≈*−46%, and *≈*−51% at 3 and 6 months; Figure 1(b)), and physician pain on a VAS (*≈*−21%, and *≈*−45% at 3 and 6 months; Figure 1(c)) improving substantially more than the prespecified effect size (20%) in patients who received polymerized collagen versus placebo (Table 2).

### 3.3. Secondary Measures of Efficacy

Scores improved considerably for patient and physician from baseline to 3 months (treatment phase) and 6 months (followup) for both active and placebo treatment (Table 2, Figure 1). There was a significant difference (*P* < 0.05) between treatments for patient's Likert score (*≈*33% and *≈*28%, at 3 and 6 months; Figure 1(g)), physician's Likert score (*≈*35% and *≈*24%, at 3 and 6 months; Figure 1(h)), patient drug evaluation (*≈*21% and *≈*48%, at 3 and 6 months), and physician drug evaluation (*≈*38% and *≈*35%, at 3 and 6 months).

### 3.4. Concomitant Medication

The placebo-treated group increased 76 per cent consumption of NSAIDs tablets per day (*P* = 0.01; Table 2, Figure 1(i)). Meanwhile, Polymerized-type I collagen-treated group decreased 83 per cent NSAIDs tablets per day (*P* = 0.016; compared baseline to final evaluation; Table 2, Figure 1(i) and *P* < 0.001 between treatments).

### 3.5. Adverse Events

The most frequent adverse event was injection site pain lasting <24 h. No patient developed aseptic acute arthritis (chemical reaction) within 24 and 72 hours after IA injection.

### 3.6. Laboratory Assessment

There were no changes in complete blood counts, measurement of liver function test, and urinalysis. Urinary CTX-II was determined as a marker of cartilage degradation. A 1.4–1.7-fold increase of CTX-II was quantified in placebo group compared with polymerized collagen group (*≈*−31% and *≈*−44%, at 3 and 6 months; Table 3). There was a statistical difference in ESR between placebo and polymerized collagen treatment (*≈*−39% and *≈*−40%, at 3 and 6 months; Table 3, *P* < 0.05).

### 3.7. Proinflammatory Cytokine Expression in T CD8 Peripheral Cells

Results show that the amounts of CD8+/IL-1*β*+-, CD8+/TNF-*α*+-, and CD8+/IFN-*γ*+-expressing peripheral cells were lower in patients under polymerized-type I collagen treatment compared with placebo at 3 and 6 months (IL-1*β*: *≈*−44% and *≈*−53%; TNF-*α*: *≈*−33% and *≈*−47%; IFN-*γ*: *≈*−34% and *≈*−12%; Table 4).

### 3.8. Proinflammatory and Anti-Inflammatory Cytokine Expression in T CD4 Peripheral Cells

The percentages of CD4+/CD14−/IL-1*β*+- and CD4+/CD14−/TNF-*α*+-expressing peripheral cells were lower in patients under polymerized-type I collagen treatment versus placebo at 3 and 6 months under treatment (IL-1*β*: *≈*−69% and *≈*−31%; TNF-*α*: *≈*−48% at 6 months; Table 4). CD4+/CD14−/IL-10+-producing cells, an anti-inflammatory cytokine, were higher in patients under polymerized collagen treatment versus placebo-treated group at 3 and 6 months (*≈*92% and *≈*32%; Table 4).

### 3.9. Proinflammatory and Anti-Inflammatory Cytokine Expression in CD14 Monocytes Peripheral Cells

**Figure 2:**
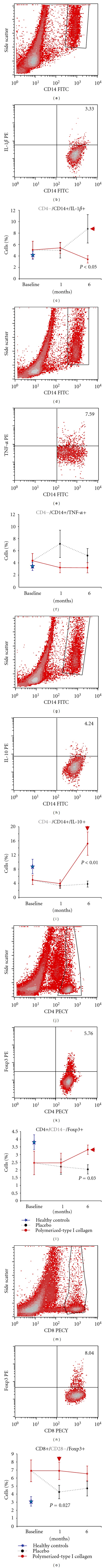
Percentage of CD14-derived proinflammatory cytokine in OA knee patients. (a) An electronic gate was made for CD4−/CD14+ single positive cells. (b) From the gate *a* CD4−/CD14+/IL-1*β*+ cells were determined. (c) Percentage of CD4−/CD14+/IL-1*β*+ peripheral blood cells was established at baseline, 3, and 6 months. (d) An electronic gate was made for CD4−/CD14+ single positive cells. (e) From the gate *d* CD4−/CD14+/TNF-*α*+ cells were determined. (f) Percentage of CD4−/CD14+/TNF-*α*+ peripheral blood cells was established at baseline, 3, and 6 months. (g) An electronic gate was made for CD4−/CD14+ single positive cells. (h) From the gate *f* CD4−/CD14+/IL-10+ cells were determined. (i) Percentage of CD4−/CD14+/IL-10+ peripheral blood cells was established at baseline, 3 and 6 months. (j) An electronic gate was made for CD4+/CD14− single positive cells. (k) From the gate *j* CD4+/CD14−/Foxp3+ cells were determined. (l) Percentage of CD4+/CD14−/Foxp3+ peripheral blood cells was established at baseline, 3, and 6 months. (m) An electronic gate was made for CD8+/CD28− single positive cells. (n) From the gate *m* CD8+/CD28−/Foxp3+ cells were determined. (o) Percentage of CD8+/CD28−/Foxp3+ peripheral blood cells was established at baseline, 3, and 6 months. The software employed was CellQuest (BD Biosciences). A total of 50,000 events were recorded for each sample. Results are expressed as mean ± SE. *P < 0.05.

Results show that the amounts of CD4−/CD14+/IL-1*β*+- and CD4−/CD14+/TNF-*α*+-expressing peripheral cells were lower in patients under polymerized-type I collagen treatment compared with placebo at 3 and 6 months (IL-1*β*: *≈*−10% and *≈*−61%, Figures 2(a)–2(c); TNF-*α*: *≈*−52% and *≈*−38%; Figures 2(d)–2(f), Table 4). 

CD4+/CD14−/IL-10+-producing cells were conspicuously higher in patients under polymerized collagen versus placebo-treated group at 3 and 6 months (*≈*18% and *≈*300%; Figures 2(g)–2(i), Table 4).

### 3.10. Regulatory T Peripheral Cells

Polymerized-type I collagen induced a statistically significant increase in Foxp3+-expressing CD4+/CD14− and CD8+/CD28− peripheral cells compared to patients under placebo treatment at 3 and 6 months (CD4: *≈*14% and *≈*65%, *P* < 0.027; Figures 2(j)–2(l), CD8: *≈*60%, *P* < 0.03 and *≈*38%; Figures 2(m)–2(o), Table 4).

## 4. Discussion

OA is the most common form of arthritis and a leading cause of disability; however, there is a large unmet need for desirable pharmacologic therapeutic interventions. This pathology offers a key research and development opportunity in terms of scientific innovation, medical requirement, and market size. The current pharmacologic agents have not been shown to have convincing disease-modifying efficacy. In this vein, combination of treatments could offer innovative therapeutic possibilities. Hence, to optimize pharmacological treatment in patients with OA, it is also necessary to assist in preserving joint integrity through arthroscopic procedures.

In this study, we determined clinical efficacy of perioperatively IA injections of polymerized collagen in symptomatic knee OA patients compared with baseline and placebo at 3 months. Clinical improvement and elevated response rates were found in primary outcomes including Lequesne Index, WOMAC pain subscale, WOMAC stiffness subscale, WOMAC disability subscale, and patient' and physician' VAS pain. Response to treatment was sustained until followup. In addition we determined improvement in secondary endpoints. We also found increased levels of urinary CTX-II only in patients treated with placebo, indicating the progression of joint damage. It is noteworthy that, patients who received polymerized collagen decreased noticeably NSAIDs consumption at statistically significant levels compared with baseline and placebo group. None of the patients have required joint replacement as of today. This clinical outcome was reached 3 to 5 months before that reported in previous polymerized collagen study [9]. This suggests that polymerized-collagen administration, after arthroscopic lavage, could improve time to response to pharmacologic therapeutics. Arthroscopic surgery removed not only particulate material, such as cartilage fragments and calcium crystals but also soluble proteins such as proinflammatory cytokines and proteolytic enzymes. On the other hand polymerized collagen downregulated pro-inflammatory cytokine production and decreased synovitis, improving motion.

However, our study has a number of limitations including the lack of X-ray analysis to determine the progression of joint space narrowing and the high rate of response to placebo. Elevated response rates to placebo have been reported in other OA trials [20–22] and may be related, at least in part, to patients' biases and expectations and to placebo administration *per se*. A similar clinical improvement in WOMAC, Patient Global Assessment, Investigator Global Assessments, and Pain compared to saline (control) injections was also observed in previous studies with the three-injection regimen of high molecular weight hyaluronan (Orthovisc) or arthrocentesis as the control [21, 22]. The most common and significant adverse events were limited to acute local reactions.

Earlier studies have shown that OA is an inflammatory disease [3–5]. Immunohistochemical studies have confirmed that synovial tissue from patients with early OA is characterized by mononuclear cell infiltration, production of proinflammatory cytokines, and mediators of joint damage. Mechanisms by which synovitis exacerbates structural damage in OA are likely to be complex. Hypotheses have included alterations in chondrocyte function, enhanced angiogenesis, changes in bone turnover, and inflammation. Excessive production of IL-1*β*, TNF-*α*, IL-8, and IL-6 by synovial tissue and cartilage can stimulate, in either autocrine or paracrine manner, chondrocytes to produce MMPs and plasminogen activator, which in turn degrade matrix proteoglycans and collagens and subchondral bone [3–8]. Besides, systemic markers of inflammation such as C-reactive protein (CRP), MMP-7, IL-15, plasminogen activator inhibitor (PAI)-1, and soluble vascular adhesion protein (sVAP)-1 are upregulated in OA as compared to controls, although to a lesser extent than that observed in RA [18, 23]. Polymerized collagen has been shown to have immunomodulatory effects on several pathologies associated with chronic inflammatory processes [8, 9, 24–27]. Hence, Polymerized collagen could be involved in downregulation of peripheral blood proinflammatory cytokine-expressing cells contributing to decrease cell activation and avoiding circulating cell migration into inflamed tissue in order to downmodulate *in situ* inflammation.

**Figure 3:**
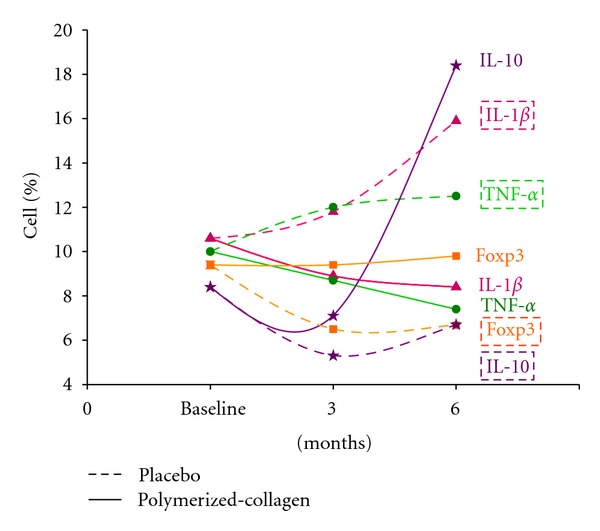
Total cytokine- and Foxp3-expressing peripheral cells in patients with symptomatic knee OA under Polymerized-type I collagen or placebo at baseline, 3, and 6 months.

Further, polymerized collagen has been shown to have a regenerator effect in experimental induction of heterotopic bone and scleroderma skin lesions [13, 28]. Our findings are in agreement with those studies, for patients under treatment with polymerized-type I collagen who showed lower percentage of IL-1*β*- and TNF-*α*-producing peripheral cells and higher number of IL-10- and Foxp3-expressing cells compared with placebo-treated group. This suggests that the inflammatory process could be modified due to downregulation of IL-1*β* and TNF-*α* production which is probably a consequence of increased IL-10 levels and higher number of Treg cells (Figure 3).

 Summing up, our results indicate that administration of polymerized type I collagen after arthroscopic lavage has an excellent safety and efficacy profile, highlighted by a low rate of injection site reactions. In addition, polymerized collagen induces systemic downregulation of inflammation. Certainly, continuing research is required to establish the potential efficacy and to increase our understanding of the biology, pharmacology, and pharmacokinetics of this biodrug.

##  Conflict of Interests

The authors declare that there is no conflict of interests.

## Figures and Tables

**Table 1:** Demographic characteristics of the patients.

	Healthy control (*n* = 13)	Placebo (*n* = 9)	Polymerized-type I collagen (*n* = 10)
		Baseline	Baseline
Age (years); Mean ± SD	42.0 ± 8.1	57.0 ± 8.1	59.9 ± 9.7
Median	42.0	53.0	56.5
Range	(40.0–54.0)	(50.0–74.0)	(50.0–75.0)
Gender (female/male)	11/2	6/3	8/2
Disease duration (years); mean ± SD		6.4 ± 3.4	4.0 ± 3.5
Median		5.0	2.0
Range		(3.0–11.0)	(0.7–10.0)
OA grade (*n*)		III (1)	III (3)
		IV (8)	IV (7)
Body mass index (kg m^−2^); mean ± SD	21.5 ± 1.6	30.7 ± 6.3	28.4 ± 3.9
Median	21.4	27.9	28.8
Range	(18.9–24.2)	(26.9–40.0)	(20.4–32.9)

**Table 2:** Clinical characteristics of the patients.

	Placebo (*n* = 9)			Polymerized-type I collagen (*n* = 10)		
	Baseline	3 months	6 months	Baseline	3 months	6 months
Patient pain (mm); mean ± SD	7.5 ± 1.2	4.8 ± 3.1	5.5 ± 2.3	7.2 ± 2.3	2.6 ± 2.5	2.7 ± 2.3
Median	8.0	4.0	5.5	8.0	1.7	2.0
Range	(6.0–9.0)	(1.0–9.0)	(1.0–8.5)	(4.0–10.0)	(0.5–7.8)	(0.0–6.5)
Physician pain (mm); mean ± SD	7.6 ± 1.5	3.4 ± 3.0	5.1 ± 2.3	7.6 ± 1.8	2.7 ± 2.9	2.8 ± 2.1
Median	8.0	2.0	4.8	8.0	1.9	2.0
Range	(5.5–9.0)	(1.0–9.1)	(1.0–8.0)	(4.0–10.0)	(0.0–8.0)	(0.0–6.0)
Lequesne; mean ± SD	13.1 ± 3.0	11.6 ± 4.0	12.9 ± 3.3	12.8 ± 2.7	7.3 ± 1.9	6.2 ± 3.6
Median	14.0	11.0	13.0	13.0	7.5	7.0
Range	(8.0–18.0)	(6.0–19.0)	(9.0–19.0)	(8.0–16.0)	(4.0–10.0)	(1.0–12.0)
WOMAC pain subscale; mean ± SD	11.2 ± 3.6	7.1 ± 3.7	8.2 ± 3.9	11.1 ± 4.1	5.8 ± 2.8	4.0 ± 3.0
Median	10.0	7.0	8.0	10.5	4.5	3.0
Range	(6.0–19.0)	(1.0–12.0)	(3.0–15.0)	(7.0–19.0)	(3.0–11.0)	(0.0–8.0)
WOMAC stiffness subscale; mean ± SD	3.9 ± 1.9	3.2 ± 2.0	3.7 ± 2.0	3.6 ± 2.6	2.4 ± 1.9	1.9 ± 1.7
Median	4.0	2.0	5.0	3.5	2.0	2.0
Range	(0.0–7.0)	(0.0–6.0)	(0.0–6.0)	(1.0–8.0)	(0.0–6.0)	(0.0–4.0)
WOMAC disability subscale; mean ± SD	37.1 ± 15.6	26.3 ± 17.2	26.4 ± 14.7	41.0 ± 14.2	19.0 ± 12.6	15.2 ± 11.6
Median	43.0	22.0	26.0	37.0	20.5	21.0
Range	(11.0–56.0)	(2.0–58.0)	(6.0–50.0)	(27.0–68.0)	(4.0–35.0)	(0.0–33.0)
Patient Likert score (cm); mean ± SD	1.1 ± 0.8	2.1 ± 1.1	2.1 ± 0.9	1.2 ± 0.6	2.8 ± 0.9	2.7 ± 0.7
Median	1.0	2.0	2.0	1.0	3.0	3.0
Range	(0.0–2.0)	(1.0–4.0)	(1.0–3.0)	(0.0–2.0)	(1.0–4.0)	(2.0–4.0)
Physician Likert score (cm); mean ± SD	1.0 ± 0.8	2.3 ± 1.0	2.5 ± 0.9	1.5 ± 0.7	3.1 ± 0.4	3.1 ± 0.6
Median	1.0	2.0	2.5	2.0	3.0	3.0
Range	(0.0–2.0)	(1.0–4.0)	(1.0–4.0)	(0.0–2.0)	(3.0–4.0)	(2.0–4.0)
Patient drug evaluation (cm); mean ± SD		2.4 ± 1.0	2.1 ± 0.8		2.9 ± 0.6	3.1 ± 0.6
Median		3.0	2.0		3.0	3.0
Range		(1.0–4.0)	(1.0–3.0)		(2.0–4.0)	(2.0–4.0)
Physician drug evaluation (cm); mean ± SD		2.1 ± 1.4	2.3 ± 1.0		2.9 ± 0.4	3.1 ± 0.6
Median		2.0	2.5		3.0	3.0
Range		(0.0–4.0)	(0.0–3.0)		(2.0–3.0)	(2.0–4.0)
Analgesic usage (tablets day^−1^); mean ± SD	40.4 ± 17.1	53.3 ± 20.0	71.1 ± 38.6	40.4 ± 17.1	13.0 ± 10.9	7.0 ± 4.9
Median	30.0	60.0	60.0	30.0	12.0	7.0
Range	(16.0–60.0)	(30.0–90.0)	(30.0–150.0)	(16.0–60.0)	(0.0–30.0)	(0.0–14.0)

**Table 3:** Laboratory characteristics of the patients.

	Placebo (*n* = 9)			Polymerized-type I collagen (*n* = 10)		
	Baseline	3 months	6 months	Baseline	3 months	6 months
Erythrocyte sedimentation rate (mmHg) Mean ± SD	15.5 ± 10.1	19.6 ± 12.9	12.8 ± 10.6	15.5 ± 10.1	11.9 ± 13.1	7.8 ± 6.7
Anti-cyclic citrullinated peptide antibodies (U); mean ± SD	1.8 ± 0.7			4.3 ± 4.8		
Median	1.9			2.7		
Range	(0.9–3.0)			(0.5–13.4)		
Urinary levels of C-terminal crosslinking telopeptide of collagen type II (ng mmol^−1^); mean ± SD	281.4 ± 291.5	283.1 ± 245.0	474.4 ± 333.9	214.3 ± 179.9	196.6 ± 167.4	266.6 ± 146.7
Median	160.0	229.3	460.4	171.9	158.9	237.4
Range	(60.1–882.8)	(102.1–896.2)	(64.6–986.0)	(6.9–592.3)	(0.1–505.9)	(91.7–472.0)

**Table 4:** PBMCs proinflammatory, anti-inflammatory cytokine production and Foxp3-expressing T cells.

	Healthy controls (*n* = 13)		Placebo (*n* = 9)		Polymerized-type I collagen (*n* = 10)	
		Baseline	3 months	6 months	3 months	6 months
CD4+/CD14−/IL-1*β*+(%); mean ± SD	1.1 ± 0.3	1.8 ± 0.3	3.5 ± 1.9	2.6 ± 0.6	1.1 ± 0.2	1.8 ± 0.4
Median	0.9	1.3	2.0	2.1	1.1	1.5
Range	(0.0–3.4)	(0.2–4.8)	(1.0–14.5)	(1.6–6.7)	(0.1–2.0)	(1.0–4.2)
CD4−/CD14+/IL-1*β*+(%); mean ± SD	3.97 ± 0.7	4.9 ± 1.5	4.8 ± 1.3	8.5 ± 2.4	5.3 ± 1.0	3.3 ± 0.6*
Median	3.7	2.4	3.9	6.9	5.8	3.1
Range	(0.8–8.0)	(0.2–18.1)	(0.8–12.8)	(1.5–18.7)	(0.6–8.1)	(0.8–6.1)
CD4+/CD14+/IL-1*β*+(%); mean ± SD	0.9 ± 0.3	0.8 ± 0.4	1.0 ± 0.3	2.9 ± 2.0	1.1 ± 0.4	1.0 ± 0.3
Median	0.8	0.7	0.6	2.1	0.7	0.8
Range	(0.1–2.1)	(0.1–3.7)	(0.3–2.5)	(1.0–6.6)	(0.2–3.7)	(0.1–2.5)
CD4+/CD14−/TNF-*α*+(%); mean ± SD	0.6 ± 0.1	1.7 ± 0.4	1.3 ± 0.4	2.3 ± 0.6	2.2 ± 0.7	1.2 ± 0.3
Median	0.6	1.5	1.0	1.5	1.5	1.3
Range	(0.0–1.9)	(0.2–4.5)	(0.2–2.7)	(0.9–5.6)	(0.5–5.5)	(0.3–2.7)
CD4−/CD14+/TNF-*α*+(%); mean ± SD	3.8 ± 0.7	4.7 ± 1.2	7.5 ± 2.3	5.6 ± 1.2	3.6 ± 0.9	3.5 ± 0.8
Median	4.1	2.4	6.9	5.3	3.4	2.7
Range	(0.6–9.5)	(0.2–17.2)	(0.3–17.2)	(1.4–9.7)	(0.7–7.2)	(0.7–8.1)
CD4+/CD14+/TNF-*α*+(%); mean ± SD	0.8 ± 0.2	0.7 ± 0.2	1.1 ± 0.6	1.2 ± 0.2	1.5 ± 0.5	0.9 ± 0.2
Median	0.7	0.5	0.3	1.1	1.3	1.0
Range	(0.1–2.1)	(0.1–2.5)	(0.2–4.6)	(0.4–2.1)	(0.3–3.7)	(0.1–2.0)
CD4+/CD14−/IL-10+(%); mean ± SD	2.3 ± 0.9	2.4 ± 0.5	1.4 ± 0.4	1.9 ± 0.5	2.7 ± 0.9	2.5 ± 0.5
Median	2.3	1.5	0.9	1.4	1.5	2.5
Range	(0.2–4.6)	(0.3–7.1)	(0.2–3.0)	(0.2–3.8)	(0.2–7.6)	(0.4–4.5)
CD4−/CD14+/IL-10+(%); mean ± SD	9.2 ± 1.1	5.0 ± 1.3	3.3 ± 0.8	3.8 ± 0.9	3.9 ± 1.1	15.2 ± 3.3*
Median	8.9	2.4	3.5	3.9	3.5	14.7
Range	(3.2–10.2)	(0.2–19.4)	(0.3–6.1)	(0.7–7.5)	(0.8–10.2)	(4.0–30.3)
CD4+/CD14+/IL-10+(%); mean ± SD	1.2 ± 0.3	0.9 ± 0.4	0.6 ± 0.2	1.0 ± 0.3	0.5 ± 0.1	1.2 ± 0.1
Median	1.1	0.4	0.5	0.6	0.4	1.2
Range	(0.2–1.6)	(0.1–6.7)	(0.0–1.6)	(0.1–2.6)	(0.1–0.8)	(0.5–1.7)
CD4+/CD14−/Foxp3+(%); mean ± SD	3.8 ± 0.5	2.5 ± 0.8	2.2 ± 0.5	2.0 ± 0.3	2.5 ± 0.6	3.3 ± 0.3*
Median	3.5	1.5	2.0	2.0	1.9	3.1
Range	(2.1–8.8)	(0.1–14.2)	(0.5–4.3)	(0.8–3.2)	(1.1–5.6)	(2.2–4.7)
CD8+/IL-1*β*+(%); mean ± SD		3.1 ± 1.1	2.5 ± 0.8	4.9 ± 1.5	1.4 ± 0.4	2.3 ± 0.5
Median		1.0	1.7	3.6	1.3	2.4
Range		(0.2–15.0)	(0.7–8.4)	(1.6–14.5)	(0.1–3.0)	(0.7–4.5)
CD8+/TNF-*α*+(%); mean ± SD		2.9 ± 0.7	2.1 ± 0.8	3.4 ± 1.5	1.4 ± 0.3	1.8 ± 0.4
Median		1.9	0.8	0.8	1.2	1.5
Range		(0.2–11.8)	(0.0–7.6)	(0.2–11.3)	(0.1–2.7)	(0.6–3.5)
CD8+/IFN-*γ*+(%); mean ± SD		7.4 ± 1.5	6.7 ± 1.6	3.7 ± 0.6	4.4 ± 0.9	3.3 ± 0.7
Median		4.6	5.6	3.0	3.8	3.0
Range		(0.5–18.8)	(1.2–17.4)	(2.2–6.8)	(0.8–8.1)	(1.1–6.9)
CD8+/CD28−/Foxp3+(%); mean ± SD	2.9 ± 0.6	6.9 ± 1.3	4.3 ± 0.8	4.7 ± 1.0	6.9 ± 1.1*	6.5 ± 1.0
Median	3.1	4.0	4.5	3.9	6.9	6.4
Range	(0.2–6.2)	(0.5–18.0)	(1.3–9.3)	(1.3–9.9)	(2.4–9.6)	(3.1–11.4)
